# Editorial: Oxidative stress and male fertility

**DOI:** 10.3389/frph.2025.1657872

**Published:** 2025-07-28

**Authors:** Roland E. Akhigbe

**Affiliations:** ^1^ Department of Physiology, Ladoke Akintola University of Technology, Ogbomoso, Nigeria; ^2^ Reproductive Biology and Toxicology Research Laboratory, Oasis of Grace Hospital, Osogbo, Nigeria

**Keywords:** oxidative stress, male fertility, testosterone, sperm, reactive oxygen species

**Editorial on the Research Topic**
Oxidative stress and male fertility

Oxidative stress is an imbalance between reactive oxygen species (ROS) generation and ROS scavenging in favor of ROS generation (1). The cells, including those in the reproductive system, are equipped with machineries that maintain optimal redox balance. These machineries are antioxidants that may be enzymatic or non-enzymatic. The enzymatic antioxidants include superoxide dismutase (SOD), catalase, glutathione peroxidase (GPx), glutathione-S-transferase (GST), glutathione reductase (GR), and catalase, while the non-enzymatic antioxidants include reduced glutathione (GSH) and vitamins such as vitamin C and vitamin E (2). On the flip side, ROS comes from endogenous (immature sperm, leukocytes, and varicocele) and exogenous sources (such as environmental toxicants: heavy metals, air pollution, pesticides, and radiation), lifestyle (stress, alcohol intake, smoking of cigarette and other substance of abuse, fatty diet, and substance abuse), infections (urinary tract infections), testicular diseases (torsion/detorsion, cancer, cryptorchidism), chronic diseases (obesity, diabetes, and hypertension), and some medications (3–7). Usually, these antioxidants feed off the generated free radicals, but sometimes, as seen in pathological conditions, the buffering capacity of the antioxidant system is overwhelmed, leading to ROS accumulation and oxidative stress.

Although ROS (at optimal levels) play significant roles in several physiological processes, they also induce pathological processes when they accumulate beyond their physiological levels. ROS act as physiological signaling molecules that are essential for the activation of the steroidogenic pathway. Mitochondrial ROS mediate the phosphorylation of extracellular signal-regulated kinase (ERK)1/2 and c-AMP-induced activation of Ras, which are critical components of Leydig cell proliferation and survival (8). ROS also promote sperm maturation by facilitating sperm DNA compaction and flagellar modifications (3). In addition to ERK1/2, ROS also promote the phosphorylation of JNK and p38 (MAPK families) in GSH-depleted cells (9). Furthermore, ROS regulate sperm capacitation, hyperactivation, acrosome reaction, and membrane fusion between the spermatozoa and the oocyte via the basification of intracellular pH, activation of cAMP-dependent signaling, removal of cholesterol from sperm membrane, and protein phosphorylation at serine, threonine, and tyrosine residues by cAMP-dependent kinases (10). In sperm-oocyte fusion, ROS activate kinases, such as PKC, and inactivate phosphatases, leading to higher enzymatic PLA2 activity that in turn breaks down fatty acids in the membrane of the sperm cells, thus increasing the fluidity of the plasma membrane (3): a key process in fertilization. On the other hand, at pathological levels, ROS induces lipid peroxidation, resulting in membrane damage and mitochondrial dysfunction of the male reproductive organs/cells (11). ROS also trigger protein oxidation and sperm DNA damage by altering the protein structure and function and promoting sperm DNA fragmentation, respectively (12, 13). ROS also induce mitochondrial and death receptor signaling, leading to apoptosis of the germ cells and other reproductive organs/cells (14, 15). Hence, oxidative stress plays a central role in male fertility. This Special Collection Frontiers | Oxidative Stress and Male Fertility provides an in-depth understanding of the roles and associated mechanisms of oxidative stress in male reproduction.

Akhigbe et al.'s Frontiers | *Viral Infections and Male Infertility: A Comprehensive Review of the Role of Oxidative Stress* reviewed the impact of viral infections on male fertility. Their review demonstrated the impact of viral infections on steroidogenesis and spermatogenesis as well as fertility capacity. Ayad et al.'s Frontiers | *Oxidative Stress and Male Infertility: Evidence From a Research Perspective* documented the sources of ROS in the male reproductive tract, assessment techniques, and the impact of ROS in male reproduction. Odetayo et al.'s Frontiers | *in vivo* *exposure to bisphenol F induces oxidative testicular toxicity: role of Erβ and p53/Bcl-2 signaling pathway* demonstrated the negative effect of bisphenol F, an analogue of bisphenol A, on testicular structure and function via the modulation of Erβ and p53/Bcl-2 signaling.

Clement et al.'s Frontiers | *Hyperhomocysteinemia in hypofertile male patients can be alleviated by supplementation with 5MTHF associated with one carbon cycle support* revealed that 5-Methyltetrahydrofolate (5-MTHF) supplementation improves hyperhomocysteinemia in hypofertile male candidates and should be considered in therapy in the management of infertility. In a single-centered pilot study, Takeshima et al.'s Frontiers | *Add-on effects of oral tocopherol supplementation to surgical varicocelectomy on the outcome of assisted reproductive technology: a single-center pilot study report* showed that oral tocopherol nicotinate after varicocelectomy shortens time to pregnancy.

Pilsova et al.'s Frontiers | *Hydrogen sulfide and its potential as a possible therapeutic agent in male reproduction* demonstrated the potential therapeutic benefit of hydrogen sulfide in male reproduction. Their study revealed that hydrogen sulfide improves sperm motility, capacitation, and acrosome reaction, exerts cytoprotective properties, and improves erectile function. Kaiyal et al.'s Frontiers | *Mitochondrial dysfunction signatures in idiopathic primary male infertility: a validated proteomics-based diagnostic approach* revealed an association of downregulated expression of PRDX5 and SOD2 in sperm samples of patients with idiopathic primary male infertility, while Yang et al.'s Frontiers | *Impact of sperm DNA fragmentation index on assisted reproductive outcomes: a retrospective analysis* showed that sperm DFI could influence embryonic development, with a higher risk of low birthweight infants in the high DFI group, but that it does not appear to affect clinical outcomes or other perinatal complications.

Kumari and Singh's Frontiers | *Tackling somatic DNA contamination in sperm epigenetic studies* suggested measures to tackle somatic DNA contamination in sperm epigenetic studies. In a systematic review and network meta-analysis, Song et al.'s Frontiers | *Effectiveness of exercise interventions on sperm quality: a systematic review and network meta-analysis* revealed the effectiveness of exercise in the management of male infertility. Outdoor aerobics improves sperm volume, while other sports enhance sperm motility and total sperm count in infertile patients. Also, resistance training enhances sperm morphology, and aerobic bicycle movement improves sperm concentration in infertile patients. Xu and Li's Frontiers | *J-Shaped relationship between the red cell distribution width to albumin ratio and erectile dysfunction: a cross-sectional study from NHANES 2001–2004* showed that red cell distribution width to albumin ratio was independently associated with ED risk, exhibiting a J-shaped relationship.

This Collection not only links oxidative stress and male fertility, but it also offers potential diagnostic and therapeutic strategies that are useful in the management of male infertility.
